# Complete genome sequences of sucrose non-fermenting non-O1/non-O139 *Vibrio cholerae* isolated from human soft tissue infection

**DOI:** 10.1128/mra.00181-24

**Published:** 2024-10-09

**Authors:** Haejin Hwang, Geraldine Salo, Melanie Orth, Nicole Podemski, Ray Erickson, Selina Jawahir, Elizabeth Cebelinski, Net Bekele, Brooke Draxler, Paul Webb, Emily Verbrugge, Julia R. Klein, Mark R. Schleiss, Evann E. Hilt, Marijke Decuir, Jisun Haan

**Affiliations:** 1 Infectious Disease Laboratory Section, Public Health Laboratory, Minnesota Department of Health, Saint Paul, Minnesota, USA; 2 Department of Pediatrics, Pediatric Residency Training Program, University of Minnesota, Minneapolis, USA; 3 Department of Pediatrics, Division of Pediatric Infectious Diseases, University of Minnesota, Minneapolis, USA; 4 Department of Laboratory Medicine and Pathology, University of Minnesota, Minneapolis, USA; 5 Foodborne, Waterborne, Vectorborne, and Zoonotic Disease Section, Infectious Disease Epidemiology, Prevention and Control Division, Minnesota Department of Health, Saint Paul, USA; University of Maryland School of Medicine, Baltimore, Maryland, USA

**Keywords:** *Vibrio*, sucrose, *Vibrio cholerae*, non-fermenting

## Abstract

This paper describes the hybrid genome assembly of sucrose non-fermenting non-O1/non-O139 *Vibrio cholerae* isolated from human soft tissue infection. The hybrid assembled genome comprises two circular chromosomes with lengths of 3,001,999 bp and 1,264,051 bp, respectively, with a G + C content of 47.38%.

## ANNOUNCEMENT

Non-O1/non-O139 *Vibrio cholerae* is associated with sporadic gastroenteritis and extraintestinal infections, including skin infection, via consumption of raw seafood and exposure to contaminated surface water (1–3). While *V. cholerae* typically ferments sucrose, sucrose non-fermenting or late-fermenting *V. cholerae* have been discovered, complicating the identification of *V. cholerae* from clinical specimens (4, 5).

The University of Minnesota Infectious Disease Diagnostic Laboratory isolated *Vibrio*-like colonies from a pustule from a pediatric case that lacked diarrheal symptoms and had recreational exposure to fresh-water sources. The initial culture was done by incubating the pustule on blood agar plate (BAP) overnight at 37°C. The isolate was sent to Minnesota Department of Health–Public Health Laboratory for further molecular characterization and sequencing.

Subsequent culturing on thiosulfate citrate bile salts sucrose agar at 36°C for 24 hours produced green colonies, indicating the absence of sucrose fermentation. Biochemical testing also confirmed the lack of sucrose fermentation. However, matrix-assisted laser desorption ionization-time of flight (MALDI-TOF) mass spectrometry testing showed the isolate as *V. cholerae* (score of 2.11). The serum agglutination test determined the serogroup of this isolate as non-O1/non-O139 (6).

The green colony was subcultured on BAP and incubated at 36°C for 24 hours for DNA extraction. Genomic DNA was extracted using QIAamp DNA Blood Mini kit on QIAcube following the manufacturer’s instructions, without DNA shearing or size selection. The same DNA extract was used for Illumina and Nanopore sequencing. The Illumina sequencing library was prepared using the Illumina DNA Prep kit #20060059. Illumina sequencing was performed on Illumina MiSeq with 2 × 250 bp paired-end reads and generated 722,820 reads. Nanopore sequencing library was prepared using the SQK-RBK004 Rapid Barcoding kit without DNA size selection or shearing and sequenced on GridION sequencer (MK1) using R9.4.1 flow cell. Reads were base called and demultiplexed using Guppy v6.3.8 with a high-accuracy base-calling mode. Nanopore sequencing produced 236,387 reads with N50 of 22,512 bp.

The reads were trimmed and filtered using Filtlong v0.2.1 (7) for Nanopore and Trimmomatic v0.33 (8) for Illumina. Prior to *de novo* assembly, the average nucleotide identity (ANI) test was performed using Illumina reads on Bionumerics v7.6. The trimmed reads were assembled *de novo* using Unicycler v0.5.0 (9), which used Illumina reads to assemble contigs and Nanopore reads to bridge gaps in contigs. The assembled contigs were annotated with PGAP 2023–10-03.build7061 (10). Benchmarking Universal Single-Copy Orghologs (BUSCO) v5.2.2 with the vibrionales_odb10 database (11) and QUAST v5.0.2 (12) were used to evaluate assembly quality and completeness. ABRicate v1.0.1 (13) was used to screen cholera toxin, thermostable direct hemolysin (TDH), and TDH-related hemolysin (TRH) genes against the Virulence Factor Database (VFDB) (14). Default parameters were used for all software unless otherwise noted.

The sequencing and annotation results are summarized in Table 1. The ANI test showed that the isolate shared 97.73% identity with *V. cholerae* 2010EL1786 (NCBI accession SRS139137). The assembled genome has a total length of 4,266,050 bp, comprising two circular chromosomes of 3,001,999 bp and 1,264,051 bp, respectively. The overall G + C content was 47.38%. The BUSCO genome completeness score was 99.7%. The annotation reported 3,819 coding sequences out of 3,959 genes. No cholera toxin, TDH, and TRH genes were detected.

**TABLE 1:** Summary of sequence data and genome features

Metric	Value
Illumina	
Total number of reads sequenced	722,820
N50 (no. of bases)	250
Genome coverage	42.36
Oxford Nanopore	
Total number of reads sequenced	236,387
N50 (no. of bases)	22,512
Genome coverage	703.89
Assembled genome	
Number of contigs	2
Total genome size (no. of bases)	4,266,050
Size of chromosome 1 (no. of bases)	3,001,999
Size of chromosome 2 (no. of bases)	1,264,051
G + C content (%)	47.38
Genome completeness by BUSCO (%)	99.72
Number of genes	3,959
Number of coding sequences (CDSs)	3,819
Number of tRNAs	105
Number of rRNAs	31

## Data Availability

The sequence reads and assembled genome have been deposited in SRA and GenBank databases under the accession numbers SRR25745292 (Illumina), SRR27991387 (Oxford Nanopore), CP145609 (Chromosome I), and CP145610 (Chromosome II; BioProject PRJNA266293, BioSample SAMN37127550, Strain identifier PNUSAV004467).
